# miR-671-5p inhibits epithelial-to-mesenchymal transition by downregulating FOXM1 expression in breast cancer

**DOI:** 10.18632/oncotarget.6344

**Published:** 2015-11-18

**Authors:** Xiaohui Tan, Yebo Fu, Liang Chen, Woojin Lee, Yinglei Lai, Katayoon Rezaei, Sana Tabbara, Patricia Latham, Christine B. Teal, Yan-Gao Man, Robert S. Siegel, Rachel F. Brem, Sidney W. Fu

**Affiliations:** ^1^ Department of Medicine (Division of Genomic Medicine), The George Washington University School of Medicine and Health Sciences, Washington, DC, USA; ^2^ Department of Statistics, The George Washington University, Washington, DC, USA; ^3^ Department of Pathology, The George Washington University School of Medicine and Health Sciences, Washington, DC, USA; ^4^ Department of Surgery, The George Washington University School of Medicine and Health Sciences, Washington, DC, USA; ^5^ Research Lab and International Collaboration, Bon Secours Cancer Institute, Bon Secours Health System, Richmond, VA, USA; ^6^ Department of Medicine (Division of Hematology/Oncology), The George Washington University School of Medicine and Health Sciences, Washington, DC, USA; ^7^ Department of Radiology, The George Washington University School of Medicine and Health Sciences, Washington, DC, USA

**Keywords:** breast cancer, miR-671-5p, tumor suppressor, FOXM1, EMT

## Abstract

MicroRNA (miRNA) dysfunction is associated with a variety of human diseases, including cancer. Our previous study showed that miR-671-5p was deregulated throughout breast cancer progression. Here, we report for the first time that miR-671-5p is a tumor-suppressor miRNA in breast tumorigenesis. We found that expression of miR-671-5p was decreased significantly in invasive ductal carcinoma (IDC) compared to normal in microdissected formalin-fixed, paraffin-embedded (FFPE) tissues. Forkhead Box M1 (FOXM1), an oncogenic transcription factor, was predicted as one of the direct targets of miR-671-5p, which was subsequently confirmed by luciferase assays. Forced expression of miR-671-5p in breast cancer cell lines downregulated FOXM1 expression, and attenuated the proliferation and invasion in breast cancer cell lines. Notably, overexpression of miR-671-5p resulted in a shift from epithelial-to-mesenchymal transition (EMT) to mesenchymal-to-epithelial transition (MET) phenotypes in MDA-MB-231 breast cancer cells and induced S-phase arrest. Moreover, miR-671-5p sensitized breast cancer cells to cisplatin, 5-fluorouracil (5-FU) and epirubicin exposure. Host cell reactivation (HCR) assays showed that miR-671-5p reduces DNA repair capability in post-drug exposed breast cancer cells. cDNA microarray data revealed that differentially expressed genes when miR-671-5p was transfected are associated with cell proliferation, invasion, cell cycle, and EMT. These data indicate that miR-671-5p functions as a tumor suppressor miRNA in breast cancer by directly targeting FOXM1. Hence, miR-671-5p may serve as a novel therapeutic target for breast cancer management.

## INTRODUCTION

The human genome is composed of approximately 1.5% protein-coding genes, with the rest being non-coding [1]. Biological functions of the non-coding genome have been widely investigated in recent years. microRNAs (miRNAs) are a class of evolutionary conserved, non-coding RNAs, 18–25 nucleotides in length, that regulate gene expression by annealing to their complementary sites on coding sequences (CDS) or 3′ untranslated regions (UTRs) of target genes [2]. Due to their stability and size, miRNAs can be readily extracted from Formalin-Fixed, Paraffin-Embedded (FFPE) samples [3], or circulating blood as stable biomarkers for cancer detection. miRNA-based anticancer therapies have recently been explored, either alone or in combination with current targeted therapies [4]. miRNAs could serve as novel diagnostic and prognostic candidates, as well as potential therapeutic targets. For example, we recently reported that miR-638 may serve as a potential novel microRNA for triple negative breast cancer (TNBC) treatment [5]. Deregulated miRNA expression profiles were identified in many human cancers [6] including breast cancer [7].

The commonly accepted model of human breast cancer proposes a linear multistep process which initiates as flat epithelial atypia (FEA), progresses to atypical ductal hyperplasia (ADH), evolves into ductal carcinoma *in situ* (DCIS), and culminates in the potentially lethal stage of invasive ductal carcinoma (IDC) [8]. This linear model of breast cancer progression has been the rationale for detection methods such as mammography in hopes of diagnosing and treating breast cancer at earlier clinical stages [9]. Breast cancer diagnosis and treatment results are currently heavily dependent on timeframe of detection and responsiveness to chemical treatment. Recent studies have shown that miRNAs could be important in breast cancer early detection as they become aberrantly expressed during tumorigenesis. Some miRNAs exhibit distinct functions in TNBC as compared to non-TNBC tumors. TNBC is known as ER-negative, PR-negative, and HER-2 negative subtype of breast cancer, which is insensitive to some of the most effective therapies available for breast cancer treatment including HER2 and endocrine therapies.

Our previous study compared miRNA expression profiles in archived microdissected FFPE components, such as normal, ADH, DCIS, and IDC, within the same tumor sample [3]. We found that one of the miRNAs, miR-671-5p, was consistently down-regulated in ADH and IDC compared to normal [3]. miR-671-5p dysfunction is associated with a few human cancers [10], but there is no report in breast cancer. Here, we identified miR-671-5p as a tumor-suppressor miRNA by targeting Forkhead Box M1 (FOXM1), an oncogenic transcription factor, in breast tumorigenesis.

## RESULTS

### Attenuated expression of miR-671-5p in breast cancer progression

**Figure 1:**
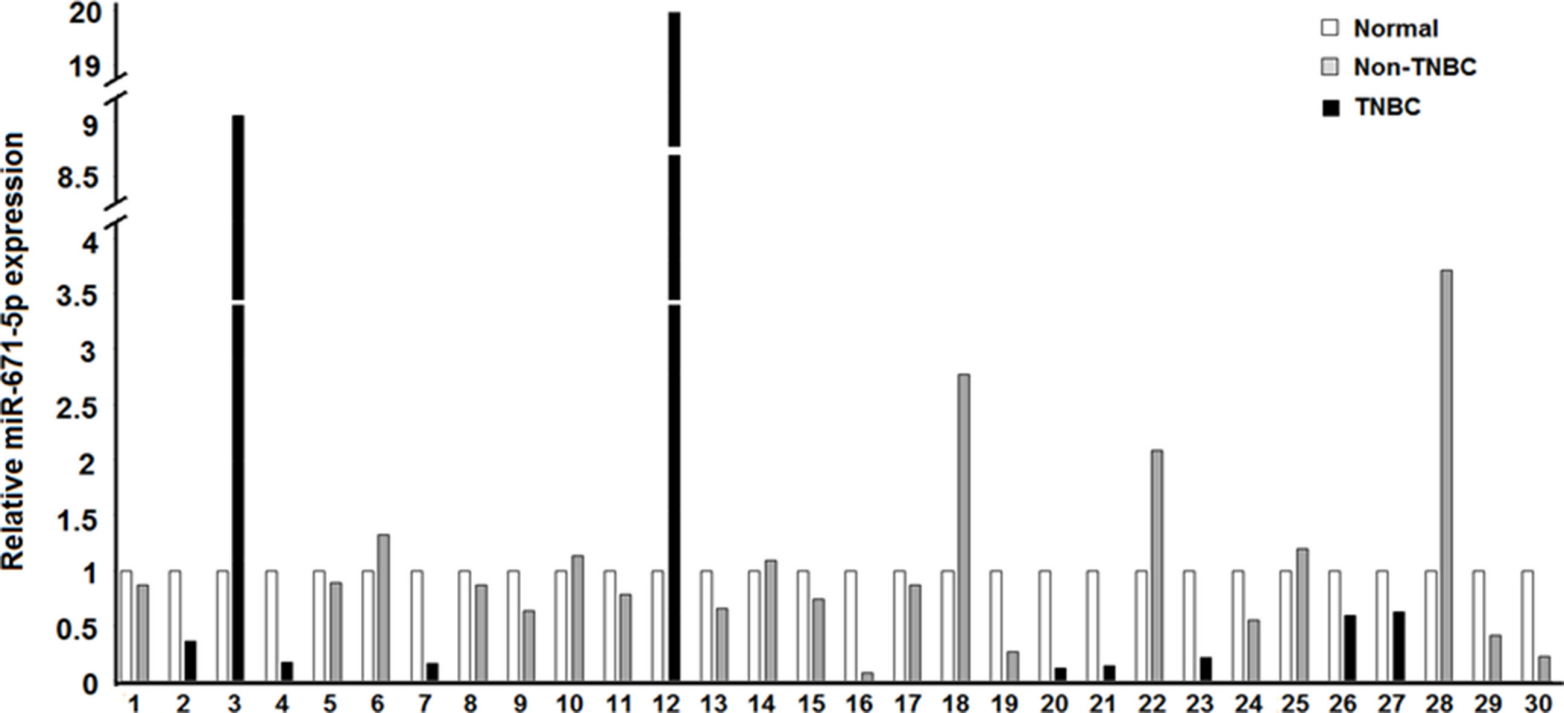
Expression of miR-671-5p in IDC vs. adjacent normal Black bars depict TNBCs while gray bars are for non-TNBCs, in comparison to the normal in white. Down-regulation of miR-671-5p expression was present in 21 of 30 (70%) of IDC compared with their adjacent normal tissue (*p* < 0.05).

In our previous work miR-671-5p was observed to be downregulated in FFPE tissues during breast cancer progression. To further investigate expression of miR-671-5p in breast cancer progression, we analyzed miR-671-5p (Acc#: MIMAT00038800) expression in a separate cohort including 30 breast cancer samples microdissected into normal and cancer cells from the FFPE tissue by qRT-PCR. Downregulation of miR-671-5p expression was present in 21 of 30 (70%) IDCs compared with their adjacent tissues (*p* < 0.05), which includes 8 of 10 TNBCs (80%) and 11 of 20 (60%) non-TNBCs (Figure 1). These results suggest the dynamic expression changes of miR-671-5p may be frequent events during the progression of breast cancer.

### miR-671-5p target gene prediction

FOXM1, a member of the FOX superfamily of transcription factors, was one of the 7304 predicted target genes of miR-671-5p by MICRORNA. FOXM1 has been implicated to play a role in cell proliferation [11], cell cycle control [12], DNA damage and repair, tumor development and progression [13], and chemotherapy [14]. As such, we chose to focus on the regulatory role of miR-671-5p on FOXM1.

### miR-671-5p regulates FOXM1 expression in breast cancer

**Figure 2:**
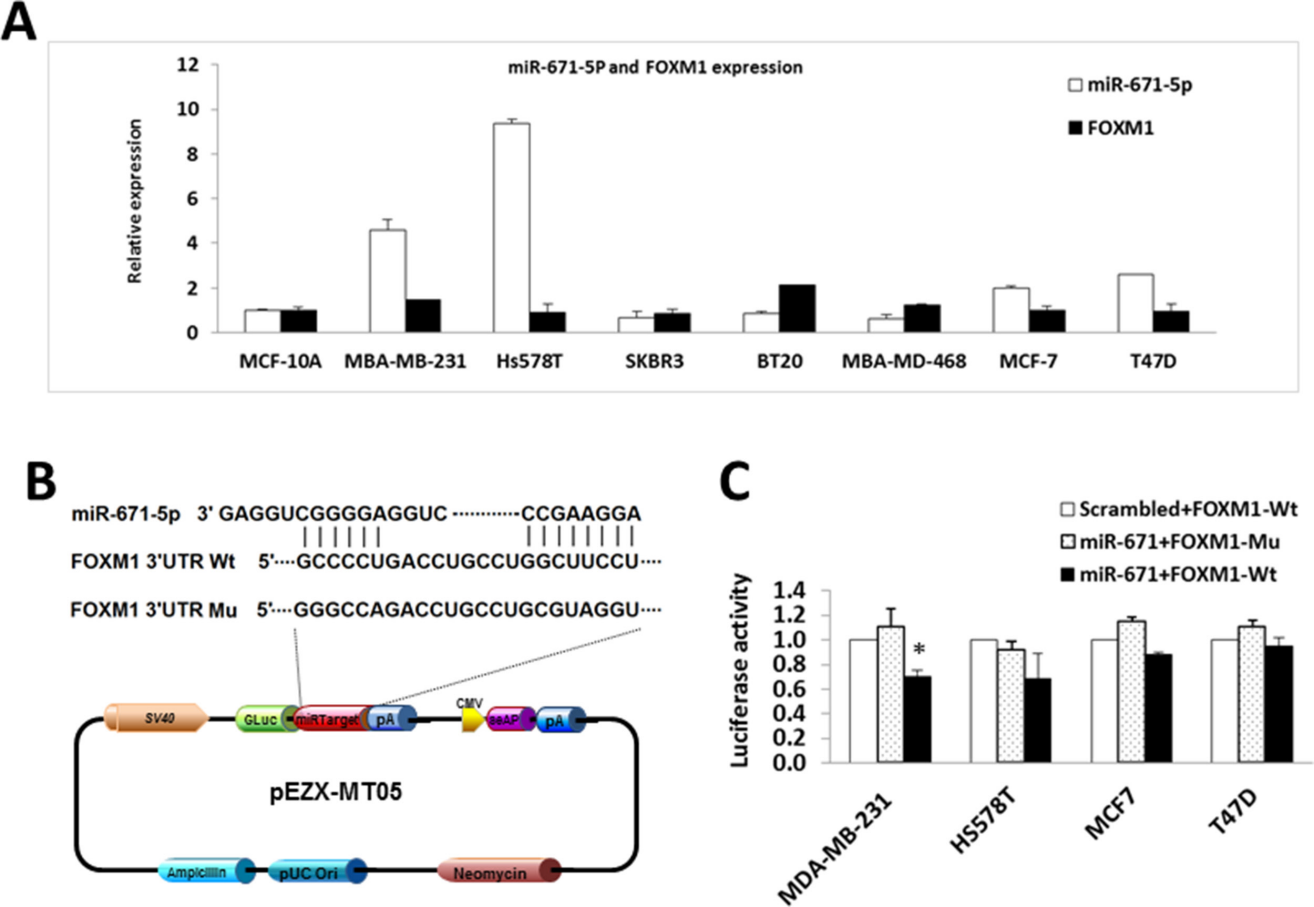
miR-671-5p targets FOXM1 in breast cancer cell lines (**A**) Inverse correlated expression of miR-671-5p and its target FOXM1 in breast cancer cell lines. (**B**) Location of the miR-671-5p binding site at the 3′-UTR of FOXM1 corresponding to the miR-671-5p sequence. (**C**) Relative luciferase activity was measured in breast cancer cell lines co-transfected with 100 ng of DNA with pEZX-miR-671-5p or pEZX-scrambled control (mock), and 100 ng of pEZX-MT05-FOXM1 (wild type or mutant) by FuGENE reagent (Promega) for 48 h. The data were reported as mean ± S.D. for three independent experiments (*p < 0.05).

To validate the computational predictions and the biological effects of miR-671-5p targeting FOXM1, we first examined the expression of miR-671-5p and FOXM1 in breast cancer cell lines. We found that the level of miR-671-5p was inversely related to FOXM1 expression (Figure 2A).

**Figure 3:**
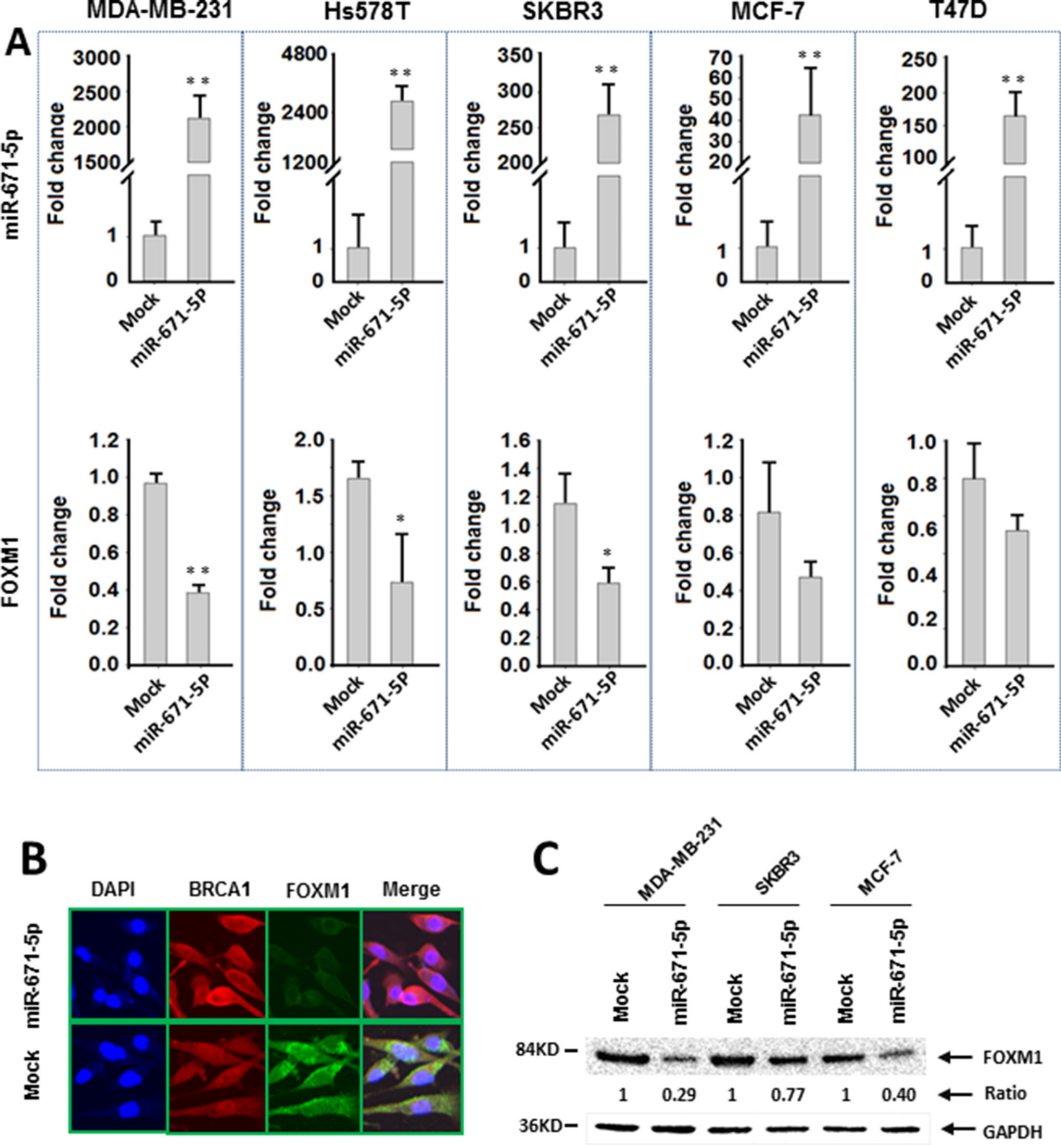
miR-671-5p negatively regulates FOXM1 expression in breast cancer cell lines (**A**) qRT-PCR analysis of miR-671-5p and FOXM1 mRNA expression in breast cancer cell lines transfected with miR-671-5p mimic or mock (**p < 0.01, *p < 0.05). (**B**) Immunofluorescence analysis of FOXM1 protein expression in MDA-MB-231 cells transfected with miR-671-5p or mock. (**C**) Western blotting analysis of FOXM1 expression in breast cancer cell lines. FOXM1 expression was decreased more significantly in MDA-MB-231 (71%) and MCF-7 (60%) cells in comparison with SKBR3 (23%) cells when miR-671-5p was overexpressed.

To confirm the specificity of miR-671-5p targeting FOXM1, we performed luciferase reporter assays with pEZX-MT05 vectors containing the miR-671-5p binding site (either wild type or mutant sequences) in the FOXM1 3′UTR region and DNA with pEZX-miR-671-5p or pEZX-scrambled control (Figure 2B). After successful co-transfection of the plasmids containing miR-671-5p and FOXM1 3′UTR wild type sequence into breast cancer cells, luciferase activities were significantly decreased in miR-671-5p transfected MDA-MB-231 cells compared with the cotransfection of those containing either miR-671-5p /FOXM1 3′UTR mutant sequence or scrambled control/FOXM1 3′UTR wild type sequence. Decreased luciferase activity was also observed in miR-671-5p/FOXM1 3′UTR wild type cotransfected in Hs578T and MCF-7 cells, although the differences were not statistically significant (Figure 2C). Our data suggest that miR-671-5p specifically targets the 3′UTR region at 828–848 nt of FOXM1 (Acc# XM_005253676.2). Consistent with the luciferase assay results, significant down-regulation of FOXM1 mRNA was observed in MDA-MB-231, Hs578T, and SKBR3 cells after overexpression of miR-671-5p. Decreased expression of FOXM1 mRNA was also observed in miR-671-5p transfected MCF-7 and T47D cells, although the change was not statistically significant (Figure 3A). Immunofluorescence staining and Western blot analyses showed decreased FOXM1 protein expression in miR-671-5p transfected cells compared with the mock control in both MDA-MB-231 and MCF-7 cell lines (Figure 3B and 3C). These results suggest that miR-671-5p directly regulates FOXM1 expression by binding to its 3′UTR in breast cancer.

### Overexpression of miR-671-5p inhibited breast cancer cell proliferation and invasion

**Figure 4:**
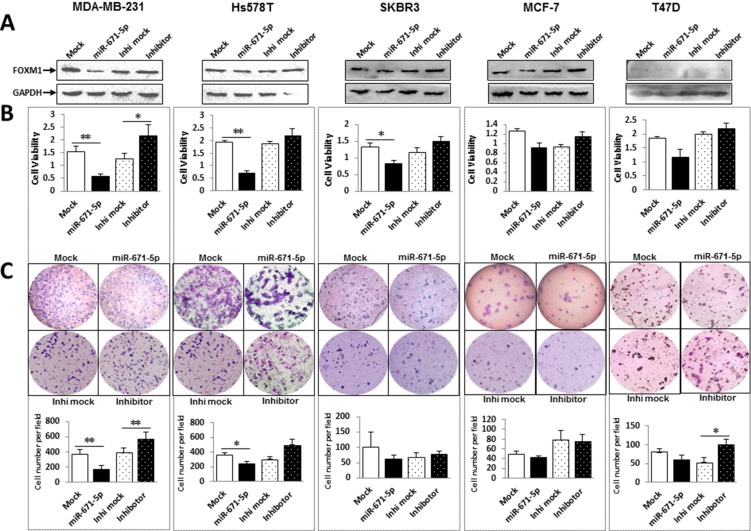
miR-671-5p inhibits proliferation and decreases invasive ability of breast cancer cell lines (**A**) FOXM1 protein expression was examined by Western blot after transfection of miR-671-5p mimic and inhibitor. (**B**) Effects of miR-671-5p on cell proliferation were determined by MTT assays. Proliferative activity was decreased after transfection of miR-671-5p mimic and increased after transfection of miR-671-5p inhibitor compared to the mock control in breast cancer cell lines. Values represent the mean ± S.D. for three independent experiments (**p < 0.01, *p < 0.05). (**C**) Transwell assays with Matrigel were performed for the invasion activity of breast cancer cells transfected with either miR-671-5p mimic or the mock control. Overexpression of miR-671-5p significantly reduces cell invasion in MDA-MB-231, Hs578T and SKBR3, but only slightly in MCF-7 and T47D. Invasion ability of the cells was displayed as a percentage of the absolute cell number (bottom). Results are displayed as mean data ± SE (**p < 0.01, *p < 0.05). Five fields of unit area on each membrane or whole membrane were counted for cell numbers, and the experiments were repeated three times in triplicate.

We have demonstrated that miR-671-5p suppressed FOXM1 expression on both the mRNA and protein levels. FOXM1 is a typical proliferation-associated transcription factor [11]. To determine if overexpression of miR-671-5p affects cell proliferation, we transfected miR-671-5p mimic to breast cancer cell lines and examined proliferation via MTT assays. Overexpression of miR-671-5p resulted in significantly reduced FOXM1 expression in protein level (Figure 4A) and decreased proliferation (Figure 4B) compared with the mock control. Conversely, transfection of miR-671-5p inhibitor resulted in increased cell growth in all cell lines. These results indicate an anti-proliferative effect of miR-671-5p in breast cancer cells.

FOXM1 has been shown to promote tumor cell invasion [15]. Using the BD Matrigel, we found that miR-671-5p overexpression exhibited significant inhibition of invasion in MDA-MB-231 cells (60%) and moderate inhibition in Hs578T and SKBR3 cells (40%) compared to the mock (Figure 4C). For non-TNBC cell lines MCF-7 and T47D the change in invasion was not as significant as in TNBC cell lines MDA-MB-231 Hs578T and SKBR3. Conversely, transfection of miR-671-5p inhibitor promoted cell growth in all cell lines except MCF-7 cells. These results suggest that miR-671-5p might play a greater role in TNBC cell lines than in non-TNBC cell lines.

### miR-671-5p induced S-phase cell cycle arrest

**Figure 5:**
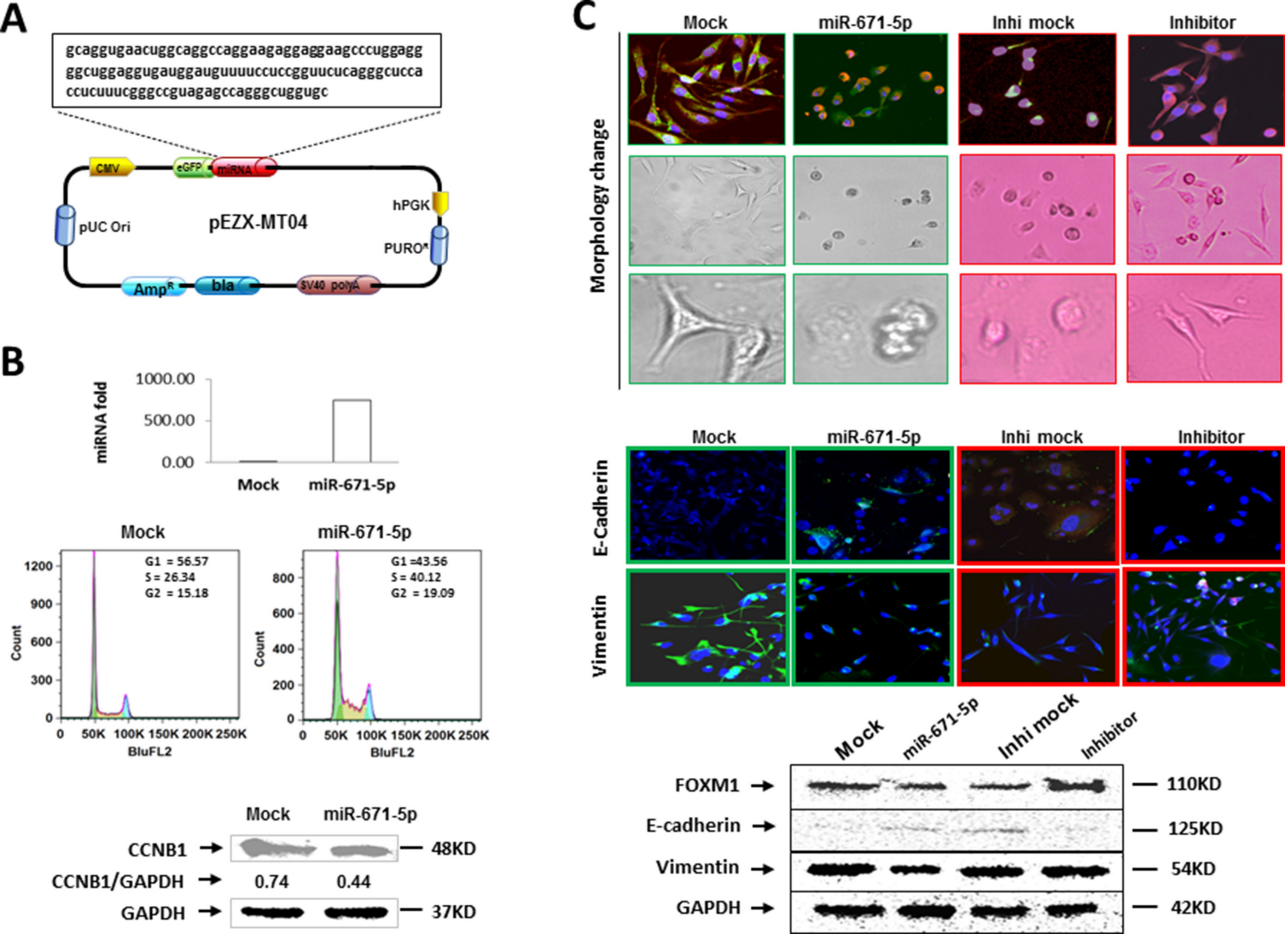
miR-671-5p induces S-phase arrest and inhibits EMT (**A**) Schematic diagram of miR-671-5p precursor sequence in pEZX-MR04. (**B**) Overexpression of miR-671-5p (top) induces S-phase arrest by flow cytometry (middle) and downregulated CCNB1 expression (bottom) in miR-671-5p stable transfected MDA-MB-231 cells when compared with mock transfected cells using Western blot analysis. (**C**) Overexpression of miR-671-5p shifts MDA-MB-231 cells from EMT to MET phenotype. Top panel showing cell morphology was observed by microscopy in MDA-MB-231 cells transfected with mock and miR-671-5p. Mock transfected MDA-MB-231 cells displayed elongated, irregular fibroblastoid morphology whereas miR-671-5p transfected cells showed a more epithelioid appearance. The middle panel shows immunofluorescence staining of E-cadherin and vimentin in the indicated cells. The bottom panel shows the Western blot analysis of E-cadherin and vimentin protein levels in indicated cells.

Due to the role of FOXM1 in cell cycle [12], we sought to examine the effects of miR-671-5p in cell cycle regulation. miR-671-5p stable transfected MDA-MB-231 cells were labeled with PI and analyzed by DNA flow cytometry. Transfection of miR-671-5p into MDA-MB-231 cells (Figure 5A) caused an S-phase cell cycle arrest and a corresponding decrease in the G_1_ phase of the cells (Figure 5B). Because CCNB1 is a downstream target of FOXM1, we investigated whether the G_2_-phase cyclin, CCNB1 [16], was affected by miR-671-5p expression. We found that CCNB1 expression was decreased in MDA-MB-231 as expected (Figure 5B and Supplementary Figure 1A). These findings suggest that miR-671-5p regulates cell cycle via FXOM1 mediated CCNB1 repression.

### miR-671-5p inhibits epithelial to mesenchymal transition (EMT)

EMT has recently been linked to cancer stem cell phenotypes [17, 18]. MDA-MB-231 cell line displays an elongated and a highly invasive, metastatic mesenchymal phenotype [19, 20]. We observed that mock transfected MDA-MB-231 exhibits the same mesenchymal shape as the parental MDA-MB-231 cells (elongated, irregular fibroblastoid morphology), while miR-671-5p transfected MDA-MB-231 cells reversed to the epithelial shape (rounded). On the contrary, a converse result was observed after miR-671-5p knockdown by transfection of miR-671-5p inhibitor in the stable transfected cells (Figure 5C, top). We next aimed to determine whether it was possible for the mesenchymal-like MDA-MB-231 breast cancer cells to undergo MET following expression of miR-671-5p. Consistent with this notion, immunofluorescence (Figure 5C, middle) and Western blot (Figure 5C, bottom) analyses revealed an upregulation of the epithelial marker E-cadherin and a concomitant reduction in the EMT marker vimentin in miR-671-5p transfected MDA-MB-231 cells. The converse E-cadherin and vimentin expression was observed with the transfection of miR-671-5p inhibitor in the stable transfected cells. These findings suggest that miR-671-5p reversed the EMT phenotype to a predominantly epithelial phenotype. Therefore miR-671-5p could be a therapeutic target for breast cancer metastasis.

### Forced expression of FOXM1 rescued cell proliferation, invasion and EMT in miR-671-5p-stable-transfected cells

To confirm miR-671-5p inhibits proliferation, invasion and EMT by directly regulating FOXM1, miR-671-5p/mock-stable-transfected MDA-MB-231 cell lines were transiently transfected with pcDNA3.1-FOXM1 or pcDNA3.1 control. After pcDNA3.1-FOXM1 transiently transfection, increased proliferation capability was detected in mock-stable-transfected MDA-MB-231, while a slight increase in proliferation was observed in miR-671-5p-stable-transfected MDA-MB-231 cells, compared to the pcDNA3.1 empty vector control (Supplementary Figure 1A). We proposed that both endogenous and exogenous FOXM1 was suppressed in miR-671-5p-stable-transfected MDA-MB-231 cells. Our data suggesting that restoration of FOXM1 abolished the inhibition of proliferation by miR-671-5p. In consistent with proliferation capability, Matrigel invasion assays revealed that restoration of FOXM1 abolished the inhibition of invasion capabilities of miR-671-5p in stable-transfected MDA-MB-231 cells (Supplementary Figure 1B). Western blotting analysis showed that overexpression of FOXM1 can restore the EMT marker vimentin and decreased epithelial marker E-cadherin in protein level by miR-671-5p (Supplementary Figure 1C). These results suggest that miR-671-5p functions as a tumor suppressor by directly targeting FOXM1.

### Overexpression of miR-671-5p sensitizes MDA-MB-231 to chemotherapy and UV treatment

**Figure 6:**
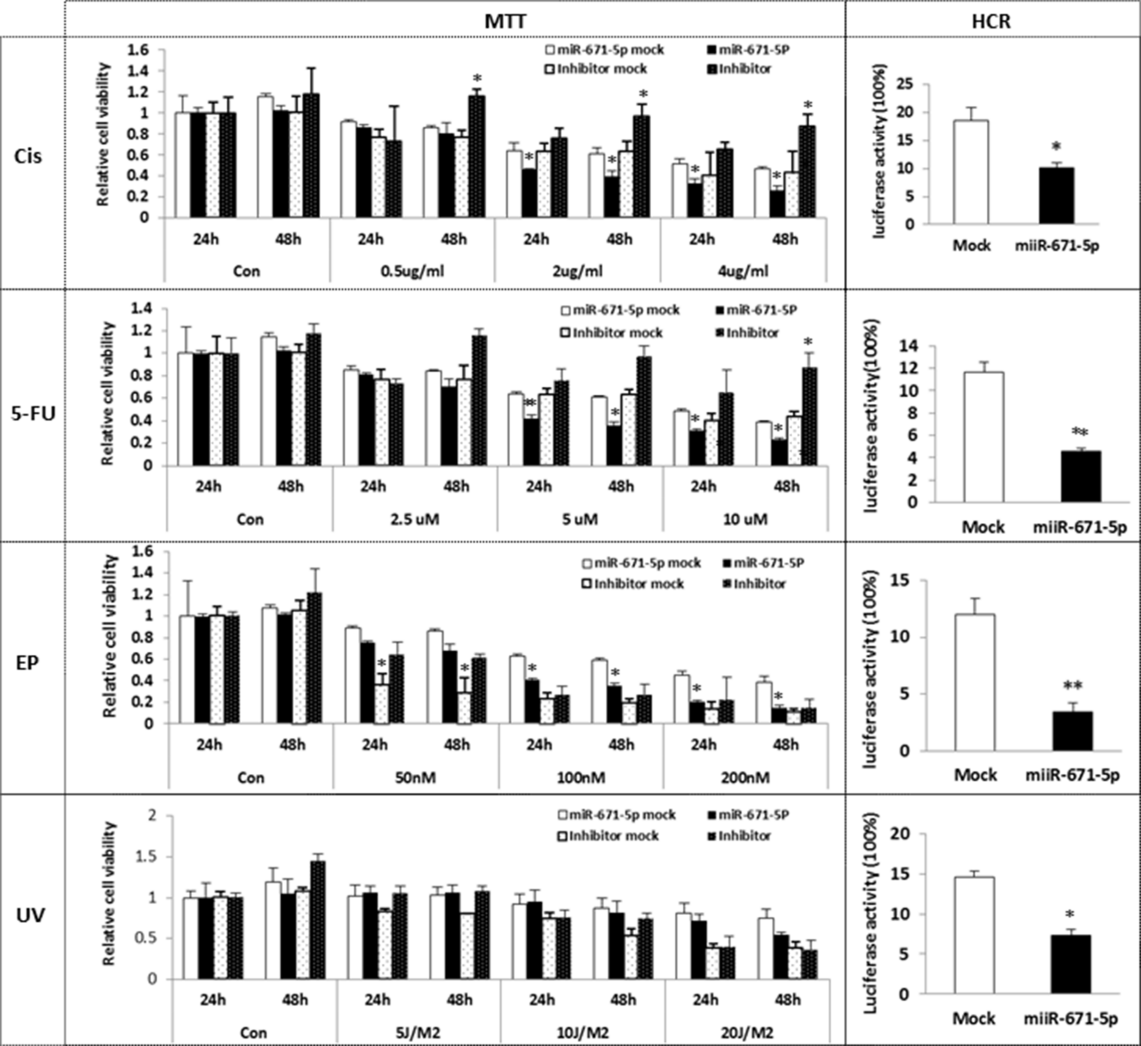
Effect of miR-671-5p on sensitivity of breast cancer cell lines to UVC/Chemosensitivity miR-671-5p or mock was stable transfected into MDA-MB-231 cell line. The stable transfected MDA-MB-231 cell line was further transfected with miR-671-5p inhibitor or mock. Cells were treated by cisplatin, 5-FU, epirubicin and UVC respectively. Cell sensitivity was measured by MTT assays. miR-671-5p overexpression significantly increased cell sensitivity to cisplatin, 5-FU and epirubicin. Results are displayed as mean data ± SE. **p < 0.01 and *p < 0.05 are considered statistically significant with comparison to the mock.

FOXM1 has been found to be overexpressed in TNBC [14] and has been suggested as a critical mediator of sensitivity and resistance to anticancer drugs, such as cisplatin, 5-FU and epirubicin [21, 14, 22]. Furthermore, FOXM1 has been reported to respond to DNA damage caused by IR (Infrared Radiation) or UV irradiation [23]. Based on our data, we reasoned that overexpression of miR-671-5p could sensitize cells to chemotherapeutic agents or UV treatment by downregulating FOXM1. To evaluate this hypothesis, we treated miR-671-5p transfected stable MDA-MB-231 cells with cisplatin, 5-FU, epirubicin, and UVC. Chemo/UV sensitivity was determined by the MTT assay. As shown in Figure 6, cells were treated with anticancer drugs and UVC in different time or dose intervals. miR-671-5p overexpression significantly increased cell sensitivity to cisplatin, 5-FU, and epirubicin in MDA-MB-231 cells compared to mock transfected lines. Repression of miR-671-5p expression by miR-671-5p inhibitor resulted in a converse effect. However, a significant sensitivity was detected only after 48 h of UV exposure in miR-671-5p transfected MDA-MB-231 cells. These results suggest that miR-671-5p might be able to reverse anticancer resistance through inhibition of FOXM1 as a new therapeutic target for breast cancer.

### miR-671-5p overexpression significantly reduced post-UV/drug host cell reactivation activity

FOXM1 has been implicated in mediating drug resistance in breast cancer by enhancing DNA repair [14]. We then asked whether miR-671-5p enhanced the sensitivity of breast cancer cell lines to chemotherapy via DNA repair pathway. We measured luciferase activity by transfecting pCMU-Luc vector, which was pre-treated by anticancer drugs and UVC, into miR-671-5p transfected stable MDA-MB-231 cells respectively. We found that miR-671-5p transfected stable MDA-MB-231 cells exhibited significantly reduced DNA repair capability compared to the mock-transfected cells. Our results demonstrate that miR-671-5p plays an important role in DNA repair by targeting FOXM1, which enhances the sensitivity of anticancer drugs in breast cancer cells.

### miR-671-5p overexpression induces global gene expression profile changes

To fully examine the effect of miR-671-5p on global gene regulation, we performed microarray analyses to identify targets experimentally by comparing miR-671-5p transfected and mock transfected breast cancer cell lines, MDA-MB-231, SKBR3 and MCF-7. The data was deposited in GEO database (Acc# GSE62411). In MDA-MB-231 cell line, a total of 81 genes were differentially expressed (Supplementary Figure 2, Supplementary Table 1; *t* test, *p* < 0.05, fold-change ≥ 1.5), including 24 up- and 57 down-regulated genes. This is consistent with our results above, where FOXM1 and CCNB1 were significantly downregulated in miR-671-5p transfected MDA-MB-231 cells compared with mock-transfected cells. In addition, genes associated with cell proliferation, invasion, cell cycle, and EMT were detected to be downregulated in miR-671-5p transfected MDA-MB-231 cells, such as GINS Complex Subunit 2 (GINS2), cyclin-dependent kinase 2 (CDK2), and minichromosome maintenance complex component 10 (MCM10) gene. In SKBR3 cell line, we found 117 genes that were differentially expressed (Supplementary Figure 1, *t* test, *p* < 0.05, fold-change ≥ 1.5), including 55 up- and 62 down-regulated. In MCF-7 cell line, 64 genes were differentially expressed (Supplementary Figure 1, *t* test, *p* < 0.05, fold-change ≥ 1.5), 40 up- and 24 down-regulated.

## DISCUSSION

MiRNAs have been shown to play important roles in carcinogenesis. Thus far only a limited number of studies have investigated miR-671-5p [10], [24, 25]. We report a previously undescribed mechanism for the tumor suppressor role of miR-671-5p in breast cancer. We showed that miR-671-5p: 1) was downregulated in breast cancer; 2) suppressed proliferation and invasion of breast cells by targeting FOXM1; 3) inhibited EMT and induced S-phase arrest; and 4) sensitized breast cancer cells to cisplatin, 5-FU and epirubicin treatment by impairing DNA repair capability. Thus, miR-671-5p appears to be a novel therapeutic target for breast cancer treatment.

### miR-671-5p serves as a tumor suppressor in human breast cancer progression by targeting FOXM1

To date, aberrant expression of miR-671-5p has been detected in hepatocellular carcinoma [25], lung cancer [10], and epithelioid sarcoma [26]. In addition, miR-671-5p has been showed to silence the *SMARCB1* expression in epithelioid sarcoma [26] and to repress *BCL2L12* expression in melanoma [24]. In our present work, we found that miR-671-5p was downregulated in breast cancer progression, and forced expression of miR-671-5p inhibited cell proliferation and invasion in breast cancer cell lines. Our results demonstrated that miR-671-5p is a potential tumor suppressor miRNA in breast oncogenesis.

**Figure 7:**
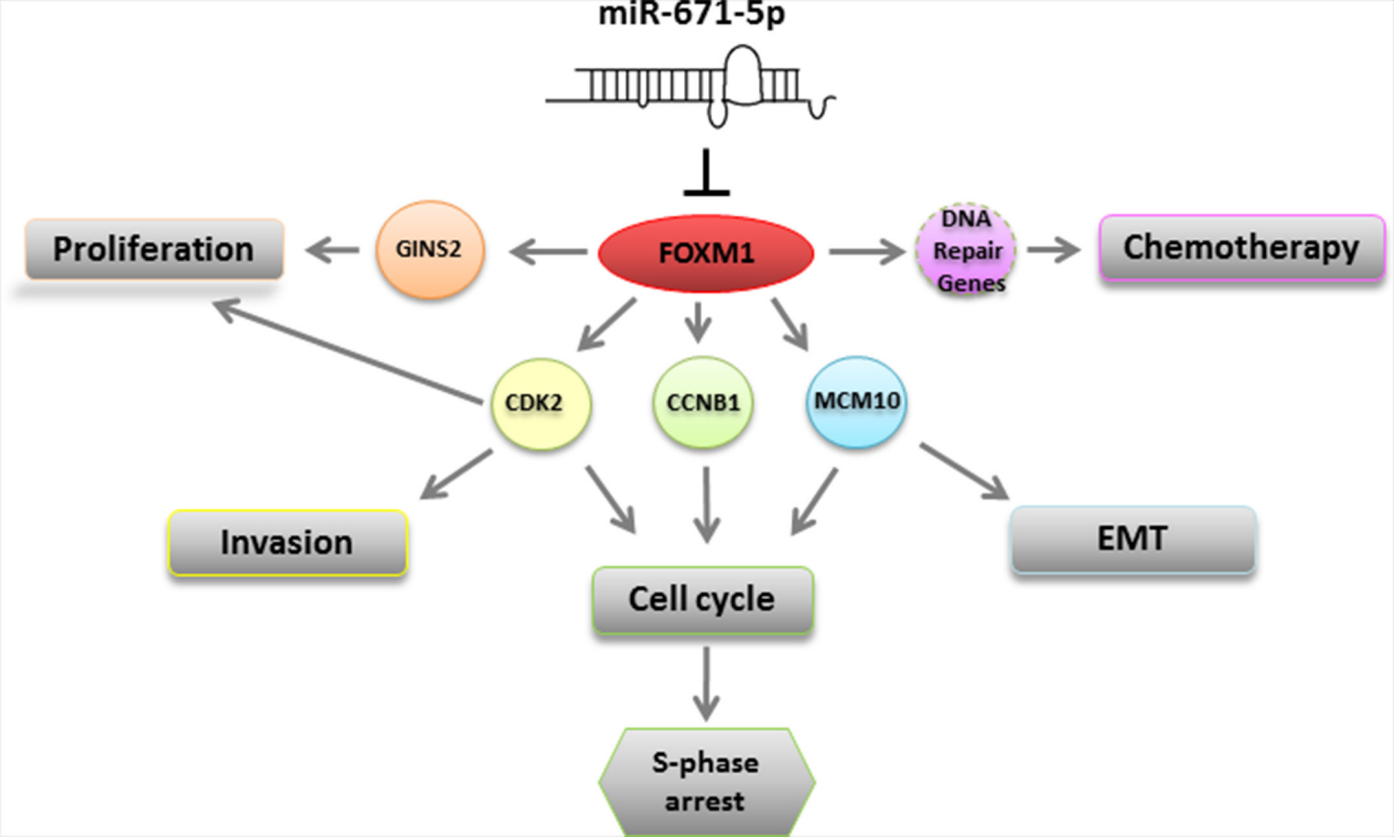
A schematic model for the regulation of miR-671-5p miR-671-5p directly targets FOXM1. Down-regulation of FOXM1 could 1) inhibit GINS2 and promote cell proliferation; 2) inhibit CDK2 and enhance cell invasion and induce S-phase arrest; 3) inhibit CCNB1 to induce S-phase arrest; 3) inhibit MCM10 which is involved in S-phase arrest and EMT; 4) inhibit CCNB1 to induce S-phase arrest; 5) affect DNA repair gene(s) to function in chemotherapy.

We showed that FOXM1 is a novel target of miR-671-5p and that overexpression of miR-671-5p can downregulate FOXM1. FOXM1 is a member of the FOX superfamily of transcription factors. FOXM1 exerts crucial role in a wide range of biological processes [27], including as a human proto-oncogene [28] involved in metastasis [29] and proliferation [30]. Unlike other Fox-transcription factors, FOXM1 is associated with cell proliferation and is expressed only in proliferating cells [31, 32]. We further found that forced expression of miR-671-5p in breast cancer cell lines resulted in FOXM1 down-regulation and significant proliferation and invasion inhibition, which implicates a tumor suppressor function of miR-671-5p by targeting FOXM1. In addition to FOXM1, our microarray data (Supplementary Figure 1) showed reduced expression of cell proliferation associated genes such as CDK2, GINS2, and MCM10 (Figure 7 and Supplementary Figure 1A) in miR-671-5p transfected MDA-MB-231 cells. We analyzed the relationship between FOXM1 and these genes by searching published data and bioinformatics information (http://itfp.biosino.org/itfp/). We found that CDK2, GINS2, and MCM10 are downstream genes of FOXM1 [33, 34]. Thus, we reasoned that the inhibited proliferation and invasion by miR-671-5p is due to suppressed FOXM1 expression and/or its downstream target genes (Figure 7).

Notably, silencing FOXM1 expression by miR-671-5p in SKBR3 and MCF-7 cells inhibited their proliferation and invasion, but not as significantly as in MDA-MB-231cells. This suggests that miR-671-5p might play a greater role in TNBC than in non-TNBC cells (Figures 3 and 4).

### miR-671-5p regulates cell cycle progression in human breast cancer cells

FOXM1 has been demonstrated as a key cell cycle regulator [27, 35, 36], which activates expression of the cell cycle genes required for both S and M phase progression [37]. However, detailed molecular mechanisms that control the level of FOXM1 during cell cycle progression remains elusive. In our study, we observed that the S-phase arrest in miR-671-5p transfected cells is associated with downregulation of FOXM1 and CCNB1. CCNB1 is essential for the initiation of mitosis. Thus, reduced CCNB1 expression could explain the ability of miR-671-5p for S-phase arrest and inhibition of G_2_-phase entry. In addition, inhibition of CDK2 has been associated with S-phase arrest [38, 39]. We detected significant downregulation of CDK2 expression in miR-671-5p transfected cells. Our data indicates that miR-671-5p may regulate cell cycle via FOXM1 mediated CCNB1 and/or CDK2 downregulation (Supplementary Figure 1).

### miR-671-5p plays an essential role in tumor suppression by inducing a shift from EMT to MET

Studies suggest that a round cellular morphology supports a less stiff cytoskeleton configuration compared with flat cellular morphology [40]. Bao *et al.* reported that overexpression of FOXM1 led to mesenchymal phenotype, while inhibited FOXM1 expression by miR-200b caused reversal of EMT phenotype in pancreatic cancer cells [41]. In our study, we observed that miR-671-5p transfected MDA-MB-231 cells showed an epithelioid appearance and expressed epithelial cell marker E-cadherin, while mock transfected cells displayed elongated, irregular fibroblastoid morphology and expressed typical mesenchymal marker vimentin, suggesting a tumor suppressive effect of miR-671-5p in EMT. It has been well documented that induction of EMT was associated with aggressive characteristics, such as cell attachment, migration, and invasion. We found that miR-671-5p transfected MDA-MB-231 cells have a significantly decreased number of invasive cells. Interestingly, we found that overexpression of miR-671-5p resulted in not only reduced expression of FOXM1, but also the MCM10 gene (Figure 7 and Supplementary Figure 1A). MCM10 is a nuclear DNA binding protein and a predicted downstream target of FOXM1. Chattopadhyay *et al.* reported that MCM10 depletion by siRNA showed a similar cell morphological change to miR-671-5p transfected cells. Furthermore, they found that MCM10 depletion inhibited proliferation and affected cell cycle progression [42]. Our data indicate that the shift from EMT to MET is due to miR-671-5p mediated downregulation of FOXM1 and MCM10. The exact mechanism warrants further investigation.

### miR-671-5p sensitized breast cancer cells to anticancer drugs

Clinically, 5-FU and epirubicin are commonly used drugs for breast cancer treatment, particularly 5-FU for TNBC [43, 44]. Cisplatin is a chemotherapeutic agent not routinely used for breast cancer treatment. However, it was reported that single-agent cisplatin could induce a response in TNBC patients [45]. Chemotherapy resistance and healthy tissue damage are major problems in cancer treatment. Therefore, identifying specific molecular targets in cancer therapy is essential. A recent study has revealed that miR-671-5p was associated with chemoradiotherapy [46]. Furthermore, FOXM1 is implicated in drug resistance of genotoxic induction, but its mechanism of action remains elusive. We found that miR-671-5p overexpression resulted in downregulated FOXM1, and further demonstrated miR-671-5p can sensitize breast cancer to anticancer drugs. Our present results implicate the potential effects of miR-671-5p on chemotherapy by regulating FOXM1.

DNA repair activity plays a critical role in therapeutic resistance. Nucleotide excision repair (NER) is responsible for the repair of bulky DNA lesions induced by UV and anticancer drugs. Cisplatin, 5-FU, and epirubicin induced DNA damage is primarily repaired via the NER [47, 48, 49]. HCR assay is applicable for the analysis of different DNA repair systems. To address the mechanism of miR-671-5p improving the sensitivity of anticancer drugs and UV irradiation, we evaluated the DNA repair activity by HCR. We observed that overexpression of miR-671-5p impaired DNA repair in breast cancer cell lines, suggesting that miR-671-5p might correspond to the cellular stress upon radiation and DNA damage agents.

Our data, for the first time, defined a role for miR-671-5p as a tumor suppressor miRNA in breast cancer, involving cell proliferation, invasion, cell cycle arrest, EMT, and chemotherapeutic sensitivity by directly targeting FOXM1 and its downstream genes. Therefore, miR-671-5p may serve as a novel therapeutic target in the management of breast cancer, particularly for TNBC.

## MATERIALS AND METHODS

### Breast cancer cell lines and cell culture

The human breast cancer cell lines, MDA-MB-231, Hs578T, SKBR3, BT-20, MDA-MB-468, MCF-7, and T47D were purchased from ATCC (American Type Culture Collection) and cultured in Dulbecco's modified Eagle's medium (DMEM) (Lonza) supplemented with 10% fetal bovine serum and 1% penicillin and streptomycin antibiotics. Immortalized MCF-10A cells were cultured in MEGM medium (CC-3150, Lonza) containing 100 ng/ml of cholera toxin to make a complete growth culture medium. All cell lines were grown in a 37°C humidified incubator with 5% CO_2_.

### FFPE breast cancer tissue microdissection

The FFPE tissue blocks were retrieved from the tissue repository of the Armed Forces Institute of Pathology (AFIP) with its IRB (Internal Review Board) approval. This study was approved by the IRB of the George Washington University. FFPE breast tissues from breast cancer patients were microdissected for the purpose of confirming the expression level of miRNA-671-5p in ADH, DCIS, and IDC as compared to normal tissue.

### Bioinformatics analysis and target prediction

The online software program from the website www.microRNA.org was used for bioinformatics analysis and target prediction of miRNAs.

### Dual luciferase reporter assay

Cells were plated (7 × 10^5^ cells/well) in 24 well plates and co-transfected with 100 ng of DNA with pEZX-FOXM1-3′UTR (wild type and mutant) expression clones inserted downstream of the firefly luciferase gene and 100 ng of DNA with pEZX-miR-671-5p or the pEZX-scrambled control (mock), using FuGENE Transfection Reagent (Promega). An independently-controlled Renilla luciferase gene was used as normalization control. Luciferase activities were determined with the Dual-Luciferase Reporter System (GeneCopoeia). Each sample was measured in triplicate using Glomax (Promega, Madison, WI). Firefly luciferase activity was normalized to Renilla luciferase expression for each sample.

### Transfection of miR-671-5p and FOXM1 in human breast cancer cell lines

Transient transfection was performed as described [5]. After overnight incubation, cells reached 30%–50% confluence, were transiently transfected with miR-671-5p mimic (Cat# 4464066), mock (mirVana™ miRNA Mimic, Negative Control, Cat# 4464059), inhibitor (Cat# 4464084), and mock (mirVana™ miRNA Inhibitor, Negative Control, Cat# 4464077) (Lifetechnologies) by Lipofectamine RNAiMAX (Life Technologies) using the Opti-MEM Reduced Serum Medium (Life Technologies). Cells were subjected for further analysis after 24 h transfection. Stable transfections were done in 6-well plates, seeded with 250,000 cells. pEZX-MR05-miR-671 containing the miR-671 precursor and pEZX-MR05-control with a scrambled sequence was obtained from GeneCopoeia (Rockville, Maryland, USA), and transfected into MDA-MB-231 cell lines using the standard FuGENE HD transfection reagent. Media containing puromycin was applied 5 days post-transfection. Stable clones were generated by plating 5 cells/ml of media in each well of a 96-well plate. For rescue experiments, the pcDNA3.1-FOXM1 plasmid containing full-length human FOXM1 was a kind gift of Dr. Huang (MD Anderson Cancer Center). miR-671-5p/mock-stable-transfected MDA-MB-231 cell lines were transiently transfected with pcDNA3.1-FOXM1 and pcDNA3.1 empty vector. Cell proliferation and invasion were examined by MTT assays and Matrigel invasion assays. EMT marker vimentin and epithelial marker E-cadherin were examined by Western blot assays.

### RNA extraction and quantitative real-time reverse transcription-PCR (qRT-PCR)

FFPE and cell line total RNAs were isolated and quantitated. miR-671-5p expression was assayed by the Taqman MiRNA Reverse Transcript Kit (Applied Biosystems), and target gene expression was analyzed using the ABI 7300 System as described previously [3]. Primer sequences are available upon request.

### Protein extraction and Western blot analysis

Proteins were extracted from cell lines using RIPA Buffer (Thermo) according to the manufacturer's protocol. Protein extraction and western blot analysis with chemiluminescent detection were as described [50]. The following antibodies and dilution factors were used: FOXM1 rabbit polyclonal antibody (13147-1-AP, 1:800, Proteintech), cyclin B1 (CCNB1) rabbit polyclonal antibody (4138P, 1:800, Cell Signaling), anti-rabbit vimentin (5741, 1:100 Cell Signaling), anti-rabbit E-cadherin (3195, 1:200, Cell Signaling), mouse GAPDH monoclonal antibody (MA5-15738, 1:2000, Sigma), anti-rabbit IgG conjugated to horse radish peroxidase (7074S, 1:2000, Cell Signaling), and anti-mouse IgG (7076S, 1:2,000, Cell Signaling).

### Immunofluorescence assays

Cells were seeded at 2 × 10^4^ cells per well on glass coverslips in six-well plates and fixed in 2% paraformaldehyde as described previously [5]. Confocal images were obtained using a LSM 510 Confocal microscope (Carl Zeiss). The number of nuclei containing at least one localized area of immunofluorescence was determined by examination of the confocal images. Antibodies for immunofluorescence assays used were as follows: FOXM1 rabbit polyclonal antibody (13147-1-AP, 1:500, Proteintech) and BRCA1 (ab16780, 1:500, Abcam), anti-rabbit vimentin (5741, 1:100 Cell Signaling) and anti-rabbit E-cadherin (3195, 1:200, Cell Signaling), Alexa Flour 568 goat anti-mouse IgG (1:500, Invitrogen), and Alexa Flour 568 goat anti-mouse IgG (1:500, Invitrogen).

### Matrigel invasion assays

Matrigel invasion assays were performed using the BD BioCoat™ Matrigel™ Invasion Chamber (BD Biosciences) as previously described [51]. Briefly, prior to the start of each experiment, 500 μl of warm (37°C) serum-free DMEM medium was added to the upper and lower chambers and allowed to rehydrate for 2 h in a 37°C cell culture incubator while 8 × 10^4^ cells were transfected by either miR-671-5p and inhibitor or their mocks and seeded onto the top chamber of pre-wetted inserts. Cells were incubated in an Matrigel chamber for 24 h. The invasive cells present were fixed, stained with the Diff-Quick staining solution and counted (five microscope fields under the 10X len). Experiments were done in duplicates for each cell line twice. Cell counts were performed on five non-overlapping random fields for each chamber and four chambers were counted for each experimental point, with the percentage of invasive cells being normalized to corresponding controls.

### Cell cycle distribution analysis

PI staining was used to analyze DNA content. Stable transfected cells with miR-671-5p were plated at concentrations determined to yield 60–70% confluence within 24 h. Cells were harvested, and labeled with PI. Briefly, cells were resuspended in PBS, fixed with 70% ethanol, labeled with PI (0.05 mg/ml), incubated at room temperature in the dark for 30 min, and filtered through 41-μm spectra/mesh nylon filters (Spectrum, Rancho Dominguez, CA). DNA content was then analyzed using the FACScan instrument equipped with FACStation running Cell Quest software (Becton Dickinson) at GW. All experiments were performed in duplicate and yielded similar results.

### Chemosensitivity and MTT assays

We first established the stable miR-671-5p or mock transfected cells, and then those cells were further transient-transfected with miR-671-5p inhibitor or inhibitor mock. Cells were washed with PBS. The MTT solution was added to each well and incubated at 37°C with 5% CO_2_ for 3 h, and removed and 100 μl DMSO was added to each well and incubated in a 37°C humidified incubator with 5% CO_2_ for 30 min. Color development was measured using a spectrophotometer at 570 nm on a plate reader (BIO-TEK Instruments) and quantified as per the manufacturer protocol (Promega, USA). To determine the drug effect after exposure, the miR-671-5p mimic or mock stable transfected MDA-MB-231 cells were seeded in 96-well tissue culture plates. Cells were treated with various concentrations of cisplatin (0.5 to 8 μg/mL), 5-Fluorouracil (5-Fu), 2.5 to 20 nM), or epirubicin (50 to 200 nM). MTT was added and absorbance was measured at different time points.

### Plasmid treatment with anticancer drugs/UV light and host cell reactivation assays

pCMVLuc reporter gene plasmid (a kind gift from Dr. Kenneth H. Kraemer, National Cancer Institute, NIH) was dissolved in 10 mm Tris-HCl, 1 mm EDTA, pH 8 (TE buffer) to a final concentration of 100 μg/ml, and poured in a Petri dish to form 1D 2 mm thick layer. For the drug treatment, 1 μl aliquots of a stock solution of 1 μg/μl cisplatin, 10 μg/μl 5-FU and 0.01 μg/μl epirubicin (Sigma-Aldrich, St. Louis, MO) in TE were added to 10 μg plasmid DNA dissolved in 200 μl TE buffer, and the samples were incubated at 37°C for the 6 h. For UV treatment, the petri dish was placed on ice and irradiated by 1000 J/m2 of UV light. DNA repair capabilities were assessed using a host cell reactivation (HCR) assay with the pCMVLuc reporter gene plasmid treated by UV or anticancer drugs [50]. Briefly, 4 μl (200 ng) of CsCl-purified pCMVLuc plasmids, damaged or non-damaged, were transfected with miR-671-5p overexpressing MDA-MB-231 cells using Lipofectamine 2000 (Invitrogen).

### Human miR-671-5p overexpression and microarray analysis

Human miR-671-5p or scrambled mock oligos were purchased from LifeTechnologies. Dilutions of 90 pmol of miR-671-5p oligos or mocks and 5 μl of Lipofectamine 2000 (Invitrogen) in Opti-MEM serum-free medium were applied to 240,000 cells in a 6-well plate. Total RNA from cells with miR-671-5p overexpressing cells were converted to cDNA and amplified by Nugen WT Applause Plus kit (Nugen). Then the cDNAs were fragmented and biotin labeled by the Encode Biotin Module (Nugen) before being hybridized to the Affymetrix Exon 1.0 ST array (Affymetrix). Data were analyzed using the GeneSpring GX 12.6.1 (Agilent). Unsupervised clustering heat map was generated on the genes with fold change > = 1.5 fold and *p* < = 0.05.

### Statistical analysis

miR-671-5p expression in clinical samples was analyzed by the exact two-sided binomial test. Data were expressed as mean ± standard error (S.E.). Permutation tests were performed for MTT assays between control and miR-671-5p mimic transfected groups. The student's *t* test (two tailed) was applied to Matrigel assay between control and miR-671-5p transfected group. *P*-values less than 0.05 were considered statistically significant.

## SUPPLEMENTARY MATERIALS FIGURES AND TABLE




