# Supplementary material for: miR-671-5p inhibits epithelial-to-mesenchymal transition by downregulating FOXM1 expression in breast cancer
Source: Oncotarget. 2015 Nov 18;7(1):293–307. doi: 10.18632/oncotarget.6344 (PMC4807999; doi:10.18632/oncotarget.6344)
Supplement: Supplementary file 1 [file oncotarget-07-0293-s001.pdf]

miR-671-5p inhibits epithelial-to-mesenchymal transition by downregulating FOXM1 expression in breast cancer

Supplementary Materials

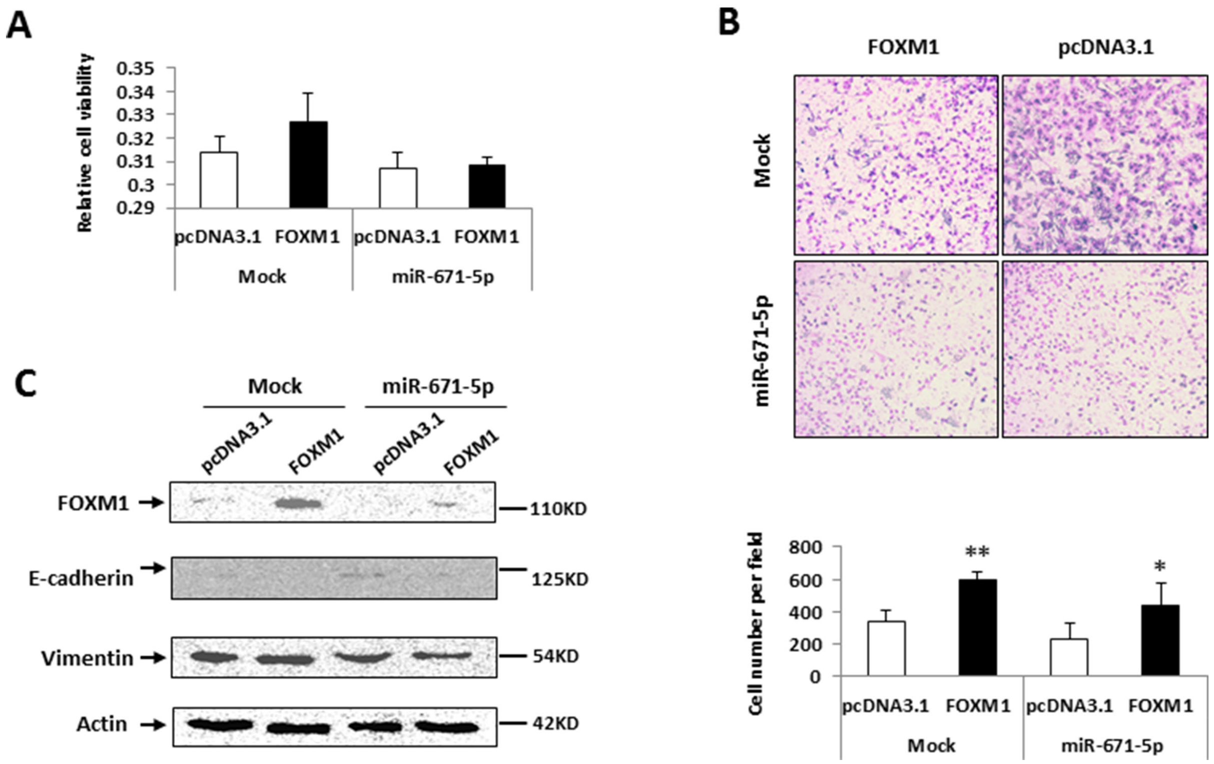

Supplementary Figure S1: Restoration of FOXM1 abolished the inhibition of proliferation, invasion and EMT. (**A**) MTT assay detected that transfection of pcDNA3.1-FOXM1 (black bars) into mock-stable-transfected MDA-MB-231 increased proliferation compared to the pcDNA3.1 empty vector control (white bars), while transfection of pcDNA3.1-FOXM1 (black bars) into miR-671-5p-stable-transfected MDA-MB-231 resulted in a slightly increased proliferation compared to the pcDNA3.1 empty vector control (white bars). (**B**) Invasion activity was examined by Transwell assays. Top panel, pcDNA3.1-FOXM1 (black bars) transfection restored cell invasion in stable-transfected MDA-MB-231 cell lines. The bottom panel, invasion ability of the cells was displayed as a percentage of the absolute cell numbers. Results are displayed as mean data ± SE. (*p <0.05 and **p <0.001). Five fields of unit area on each membrane or whole membrane were counted for cell numbers, and the experiments were repeated three times with triplicates. (**C**) Western blot analysis of E-cadherin and vimentin protein levels in indicated cells.

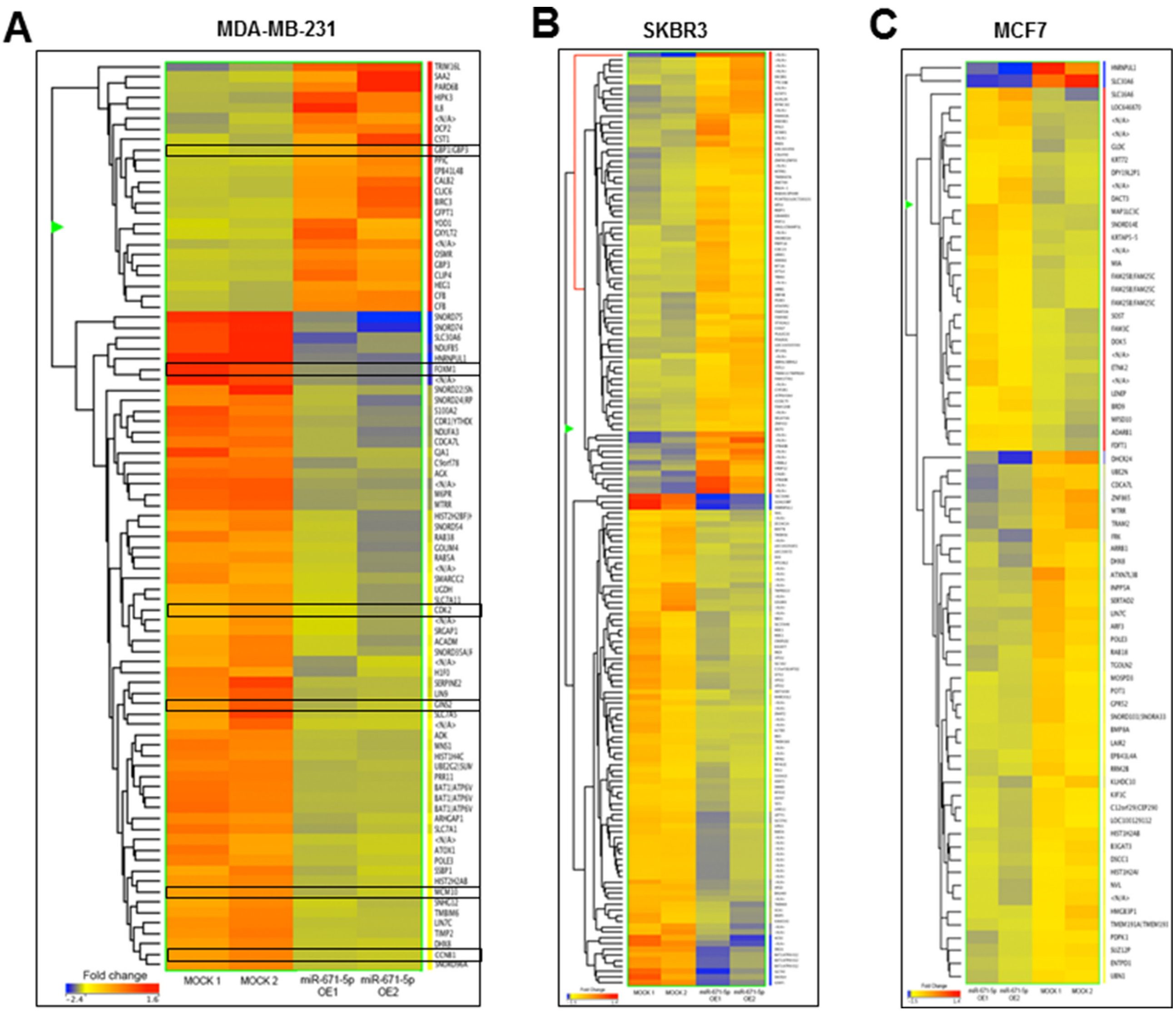

Supplementary Figure S2: Gene expression profile of target genes of miR-671-5p in breast cancer cell lines, MDA-MB-231. (**A**), SKBR3 (**B**) and MCF-7 (**C**) by Affymetrix Human Exon 1.0 ST arrays. Expression data were mean centered and the relative value for each sample is represented by color, with red representing high expression and blue representing low expression.
